# Adjuvant Scrotal Radiation Therapy As Bridging Therapy to Chimeric Antigen Receptor T-Cell Following Extramedullary Relapse in B-Cell Acute Lymphoblastic Leukemia

**DOI:** 10.7759/cureus.20134

**Published:** 2021-12-03

**Authors:** Colton Ladbury, Amandeep Salhotra, Savita Dandapani

**Affiliations:** 1 Radiation Oncology, City of Hope National Medical Center, Duarte, USA; 2 Hematology and Hematopoietic Cell Transplantation, City of Hope National Medical Center, Duarte, USA

**Keywords:** scrotal irradiation, b-all, bringing radiation, car t, leukemia

## Abstract

Chimeric antigen T-cell (CAR T) therapy is a promising emerging treatment option for patients with relapsed/refractory acute lymphoma. The role of bridging radiotherapy prior to CAR T infusion is an area of increasing interest with a sizable body of literature regarding its use in non-Hodgkin lymphoma, but reports of its use in leukemia are limited. Furthermore, available literature on bridging radiotherapy is limited to the treatment of bulky, often symptomatic disease, as opposed to its role in treating high-risk regions and sanctuary sites. Here, we present an adult male with multiply relapsed B-cell acute lymphoblastic leukemia (B-ALL) who presented with bone marrow relapse and extramedullary relapse in the right testicle. He was successfully treated with right orchiectomy followed by adjuvant bridging radiotherapy to the left testicle and scrotum, followed by CAR T infusion. Under this treatment paradigm, he tolerated the CAR T infusion with minimal toxicity and was without evidence of disease 100 days post-infusion, with normal testosterone levels. This is the first reported case of bridging radiation being used in the adjuvant setting in a patient with hematologic malignancy. This case adds to the growing body of literature that bridging radiation is well-tolerated and can potentially decrease the risk of relapse in high-risk areas following CAR T infusion.

## Introduction

Cluster of differentiation (CD)19-directed chimeric antigen receptor T-cell (CAR T) therapy is a promising emerging therapy in the setting of relapsed/refractory acute lymphoblastic leukemia in both pediatric and adult patients [1,2]. However, in most cases, bridging therapy is required during the cell manufacturing process (approximately four weeks) between leukapheresis and CAR T infusion due to extent of active disease and concern for rapid progression and associated symptomatic disease, with preference given to agents that are not CD19 directed due to concern for antigenic loss [3]. Bridging therapy can include systemic therapies such as steroids, chemotherapy, targeted agents (blinatumomab, inotuzumab), or radiation therapy. There is limited evidence for bridging radiation being used in leukemia, with available literature limited to cases of cardiac and orbital involvement in pediatric B-cell acute lymphoblastic leukemia (B-ALL) [4,5]. Here, we present a patient with multiply relapsed B-ALL with extramedullary recurrence in the testicle that was subsequently treated with orchiectomy and prophylactic bridging radiotherapy to the contralateral testicle and scrotum, which helps enable an effective administration of CAR T therapy with excellent response.

## Case presentation

A 24-year-old male was initially diagnosed with Philadelphia chromosome-negative B-ALL with 60% lymphoblasts present in hypercellular marrow. The blasts were positive for CD34, terminal deoxynucleotidyl transferase (TdT), CD22, CD38, CD19, and CD10, and cytogenetics revealed a 46XY karyotype. Fluorescence in situ hybridization (FISH) testing confirmed KMT2A (MLL2)/11q23 gene rearrangement. He received induction chemotherapy with a pediatric-inspired regimen and achieved morphologic remission minimal residual disease (MRD) positive. He subsequently underwent allogenic transplant using double umbilical cord blood from a matched unrelated donor after myeloablative conditioning (Cytoxan, fludarabine, thiotepa) and total body irradiation to a total dose of 400 cGy in two fractions.

Four years post-transplant, the patient developed fevers of unknown origin, tiredness, and fatigue in addition to bone pains. A differential blood count revealed the presence of blasts in the peripheral blood, with relapsed disease confirmed with bone marrow biopsy revealing hypercellular marrow with blasts continuing to be positive for CD19. Next-generation sequencing (NGS) confirmed MLL/KMT2a gene rearrangement. He initially received salvage treatment with blinatumomab for CD19 positive B-ALL relapse but had refractory disease after one cycle of treatment. He was subsequently enrolled on a research protocol using inotuzumab ozogamicin with cyclophosphamide-vincristine-prednisone (CVP) chemotherapy and again achieved minimal residual disease (MRD) negative remission after one cycle of treatment. He then received consolidation with haploidentical transplant with reduced-intensity conditioning with melphalan, fludarabine, and a single dose of total body irradiation (TBI) at 200 cGy.

**Figure 1:**
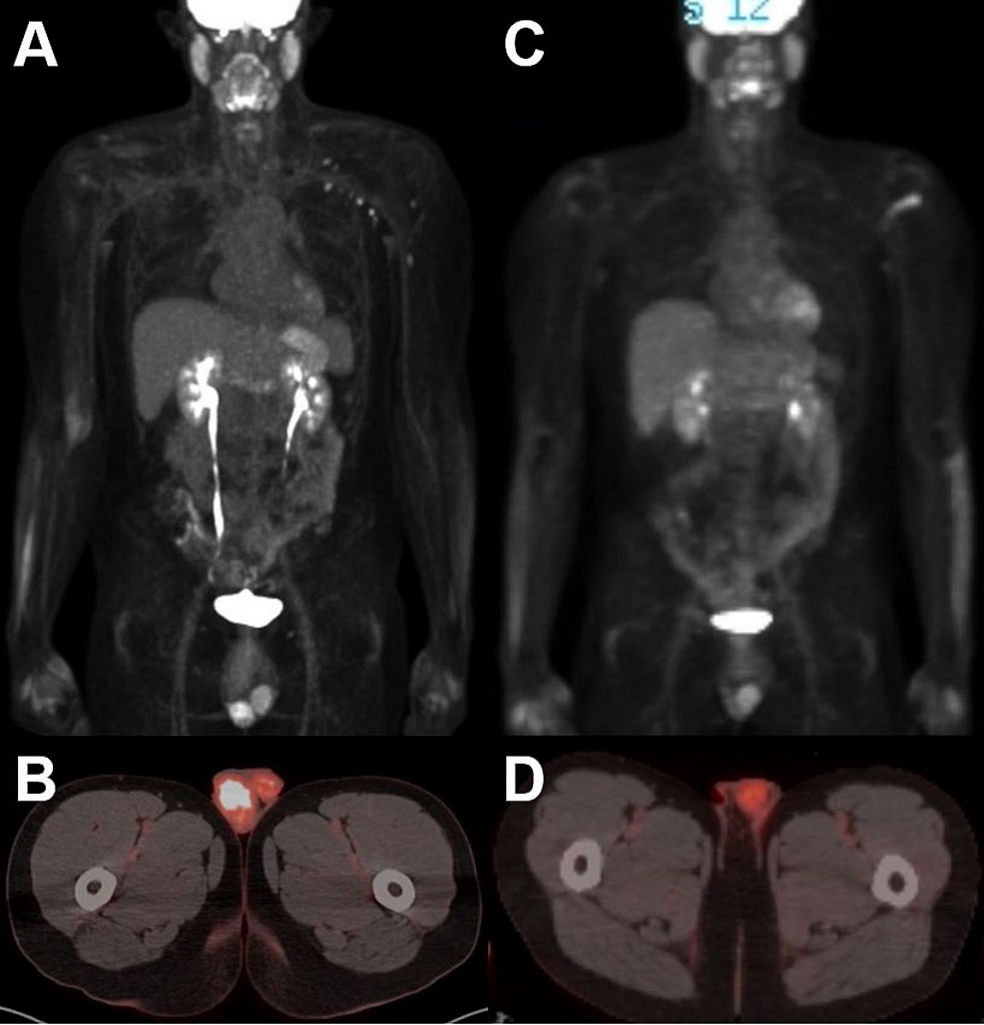
FDG PET-CT prior to orchiectomy and bridging radiation therapy (A and B), and after orchiectomy and bridging radiation therapy (C and D) FDG: fluorodeoxyglucose; PET: positron emission tomography; CT: computed tomography

The patient ultimately relapsed again three years later with systemic (MRD positive) and extramedullary relapse, this time with a right testicular mass. At the time, bone marrow biopsy revealed normal karyotype with 0.66% blasts that were positive for the following markers: CD19, CD22 (dim), CD24, CD34, CD38 (dim), and human leukocyte antigen-DR isotype (HLA-DR). Lumbar puncture was negative. Positron emission tomography-computed tomography (PET-CT) revealed a fluorodeoxyglucose (FDG) avid right testicular mass and no other sites of disease including the left testicle (Figure 1, panels A and B). Due to his multiply relapsed disease, refractory to prior chemotherapies and transplant, CD19-directed CAR T therapy was recommended. However, in the setting of known testicular disease, it was recommended that he undergo orchiectomy for further evaluation prior to CAR T.

**Figure 2:**
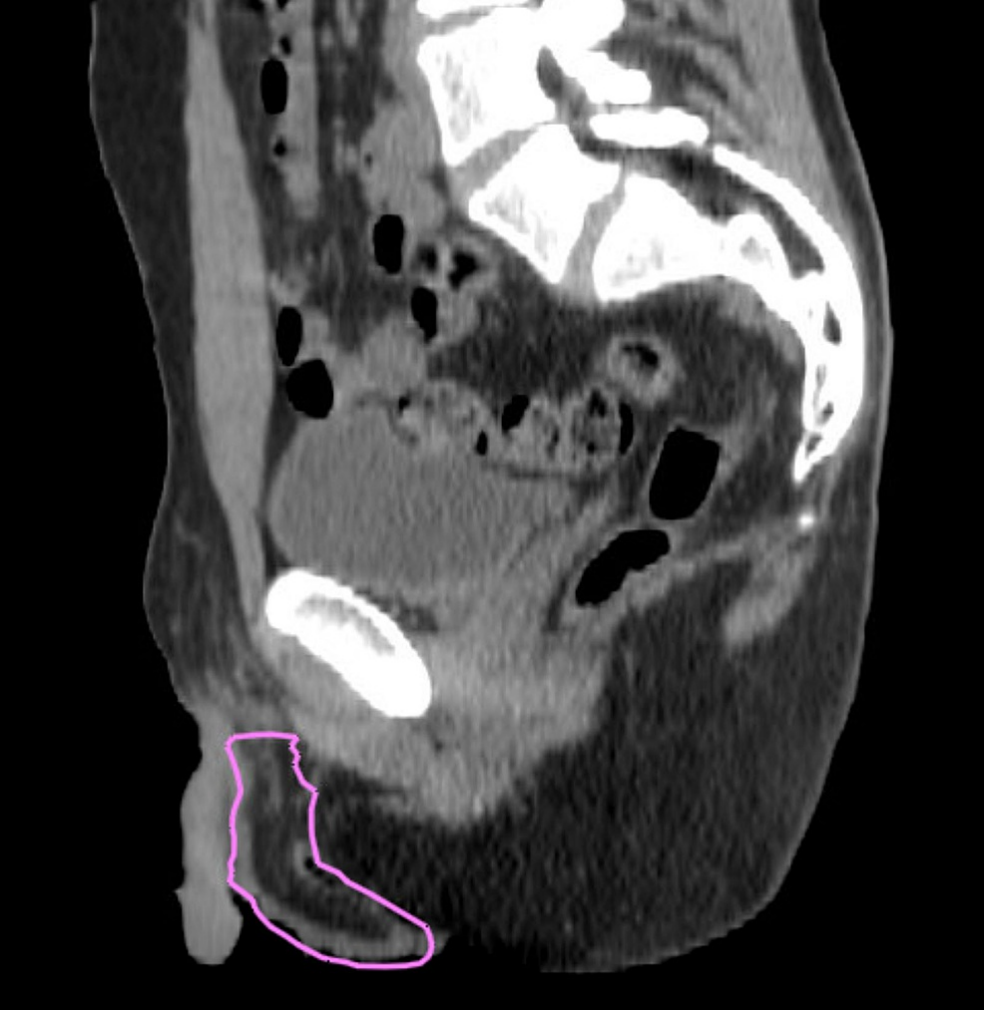
Radiation treatment volumes CT sagittal view of radiation target volumes treated with clinical setup. Pink line represents the clinical target volume. CT: computed tomography

The patient underwent right radical orchiectomy, with pathology consistent with B-cell ALL. NGS testing revealed KMT2A gene rearrangement in testicular mass as well. He was therefore recommended adjuvant radiation to the contralateral testicle and scrotum for improved local control and as a bridge to CAR T, given that the testicle is a sanctuary site and the patient was not offered systemic therapy for MRD positive disease in bone marrow. Following leukapheresis, radiation was administered to the scrotum and contralateral testicle using 12 eV enface electrons to a total dose of 2400 cGy in 12 fractions, the standard dose in the COG AALL0331 trial (Figure 2). Radiation was well-tolerated with grade 1 radiation dermatitis. PET-CT performed following his treatment was negative for evidence of malignancy, with no change in uptake in the left testicle (Figure 1, panels C and D).

Two weeks after completing radiotherapy, he received lymphodepleting chemotherapy with fludarabine/Cytoxan followed by CD19-directed CAR T infusion. On day +6 post-CAR T cells, he developed grade 2 cytokine release syndrome (CRS) which was treated conservatively without steroids or IL-6 directed therapy without evidence of neurotoxicity. He did not experience any tumor lysis syndrome. He was discharged without significant complication on day +14 post-CAR T. Bone marrow biopsy on day +100 post-CAR T therapy demonstrated MRD negative complete remission. Restaging PET/CT, MRI of the brain, and lumbar puncture were all also negative. As of the last follow-up at six months, the patient remains without evidence of disease. In addition, the patient’s testosterone levels are still in the normal range without the requirement for testosterone injections. 

## Discussion

As with other hematologic malignancies, CAR T-cell therapies have rapidly joined the most promising treatments for relapsed or refractory (R/R) leukemia. In one phase I/IIA trial in children and adults, six-month event-free survival and overall survival rates were 68% and 78% [6]. However, patients with higher disease burden going into CAR T infusion raise concerns both for increased risk of relapse as well as increased incidence of CAR T-related toxicity, not to mention the obstacle symptomatic higher disease burden poses to patients even getting to infusion [6-8]. Bridging therapy, in the form of systemic therapies or radiation, is therefore used to try to mitigate these barriers and concerns [9].

Radiation therapy (RT) provides many possible benefits as a bridging modality. There is evidence that it can aid with cytoreduction, lymphodepletion, treatment of sanctuary sites, and enhancement of the immune response [10,11]. Further, preclinical data suggests it can mitigate antigen-negative tumor relapse and potentially promote susceptibility to CAR T-cell therapy via tumor necrosis factor-related apoptosis-inducing ligand (TRAIL)-mediated apoptotic death [12]. A current theory is that RT might also promote migration of CAR T cells to the tumor site and increase effector functions [13]. Therefore, in total, bridging RT may act on multiple facets of the CAR T mechanism, ultimately improving the patient response.

There are limited data on the use of bridging radiotherapy prior to CAR T infusion for leukemia, although one prior case report demonstrated its utility in controlling bulky extramedullary cardiac and gastric disease [4] while another provided local control to ocular disease [5]. However, there is a growing body of retrospective studies on the use of bridging therapy for R/R large B-cell lymphoma, with favorable toxicity profiles and oncologic outcomes [14-16]. In the largest of these studies by Pinnix et al., which included 11 patients who got radiation bridging alone and six patients who got combined radiation and systemic therapies having no significant difference in cytokine release syndrome (CRS) or neurologic toxicity relative to patients who did not receive bridging therapy or who received systemic bridging therapy alone, although numerically toxicity rates were more favorable in patients who got RT [15]. In this study, objective response rate (ORR) and complete response rate (CRR) were 100% and 82%, respectively, in the RT group, relative to an ORR and CRR of 67% and 38% in the chemotherapy group. Median progression-free survival and overall survival were significantly higher in the RT group compared to the systemic therapy group at 8.9 months vs 4.7 months and not reached (NR) vs 21.9 months, respectively. The results of the other available studies are comparable to these ones, and in total suggest RT may help limit toxicity and prime treatment responses to CAR T [17]. However, it is worth noting that in these studies radiation was given to patients with more limited sites of disease which is a confounding factor worth considering.

This happens to be true for the patient in our case, given that at the time of relapse the only known sites of disease were the right testicle and low-level disease (MRD positive) bone marrow. However, this case is distinct from available bridging RT literature in that the target volumes did not contain the known disease and was not given to decrease tumor bulk, but rather was given adjuvantly with the goal of decreasing future failure in a sanctuary site of high clinical risk. In this patient, there was concern that systemic therapies would have decreased availability in the uninvolved testicle due to the blood-testis barrier, although this impact is a matter of controversy and the impact it could have on CAR T function is not well-defined [18]. Regardless, in the setting of unilateral testicular relapse, prophylactic radiation following orchiectomy is recommended, irrespective of whether CAR T is planned, given limited data for CAR T when patients have testicular relapse [19]. Data on contralateral testicle failure is limited, but in one small case report of patients with isolated testicular relapse, three of seven patients treated with orchiectomy without adjuvant radiation subsequently relapsed in the testicle [20]. Our patient also had bone marrow disease and had multiply relapsed disease, so concern for subsequent relapse in the scrotum was high. This was particularly true given his KMT2A rearrangement, which has been shown to have overall poor prognosis and is characterized by aggressive relapse [21]. Therefore, offering bridging RT as a means of controlling his highest risk site for extramedullary (EM) relapse post-CAR T, while simultaneously augmenting the CAR T response shows how such treatment may be beneficial even without measurable disease on imaging.

## Conclusions

As CAR T therapy for hematologic malignancies, including acute leukemia, continues to increase, it will become increasingly important to determine how to optimize treatment and limit recurrences in patients with bulky disease or areas at high risk for disease prior to CAR T infusion. This is the first report of adjuvant bridging RT targeting an area of high risk for disease prior to CAR T infusion in ALL or any other hematologic malignancy, as well as the first case of bridging RT for acute leukemia in an adult patient, and adds to the growing body of literature that radiation administered between leukapheresis and CAR T infusion is well-tolerated and may aid in producing more favorable oncologic outcomes.
